# ﻿Taxonomy of *Homoeusa* Kraatz, 1856 (Coleoptera, Staphylinidae) from the East Palearctic: I. *Homoeusarufescens* (Sharp, 1874) and a new allied species

**DOI:** 10.3897/zookeys.1121.85489

**Published:** 2022-09-12

**Authors:** Tsubasa Nozaki, Munetoshi Maruyama

**Affiliations:** 1 Entomological Laboratory, Graduate School of Bioresource and Bioenvironmental Sciences, Kyushu University, Fukuoka 819-0395, Japan Kyushu University Fukuoka Japan; 2 The Kyushu University Museum, Fukuoka 812-8581, Japan The Kyushu University Museum Fukuoka Japan

**Keywords:** Aleocharinae, description, Dinardina, *Homoeusaovata* sp. nov., *Lasius*, myrmecophily, new species, rove beetle

## Abstract

There is insufficient information to identify most species of the myrmecophilous rove beetle genus *Homoeusa*. In this paper, after examining the type material, *Homoeusarufescens* (Sharp, 1874) is redescribed in detail and its new allied species *Homoeusaovata***sp. nov.** is described. We also observed the behavior of these two species in the field; the behavior was similar to that reported for *H.acuminata* (Märkel, 1842). A checklist of *Homoeusa* from the Palearctic and Nearctic is also provided.

## ﻿Introduction

Nine species of the myrmecophilous genus *Homoeusa* Kraatz, 1856 (tribe Oxypodini) are known from the Palearctic region, of which eight are recorded from the East Palearctic, i.e., China, Taiwan, Far East Russia, and Japan (Smetana 2004; Pace 2010; Maruyama et al. 2013). Most species have not been redescribed since their original descriptions 90–120 years ago, which are very short and almost useless for identification. This series of papers aims to revise the East Palearctic species of *Homoeusa*.

*Homoeusa* is distinguishable from the other myrmecophilous oxypodine genera of the Palearctic by its sub-limuloid body shape and unilobed ligula; the latter distinguishes it from the similar genus *Thiasophila* Kraatz, 1856, which has a bifid ligula. Maruyama and Zerche (2014) suggested that *Thiasophilarufescens* Sharp, 1874 described from Japan could be a member of *Homoeusa*. Probably based on this statement, Schülke and Smetana (2015) listed *T.rufescens* as a *Homoeusa* species. Here, we confirmed that *T.rufescens* should be transferred to *Homoeusa*. We found that another species allied to *T.rufescens* is included in the syntypes of *T.rufescens* and is very common in the nests of *Lasius* species in the *fuliginosus* group (formerly members of the subgenusDendrolasius). It was found to be a new species and is also described here. The taxonomy of *Homoeusa* is very difficult and some species complexes still require time to work. Therefore, this paper treats these two species, which are very common and need to be identified, prior to the revision of the whole genus. We discuss the feeding behavior of these species and provide a checklist of the world species of *Homoeusa*.

## ﻿Materials and methods

The material examined in this study is deposited mostly in the Kyushu University Museum (**KUM**), and some in The Hokkaido University Museum, Sapporo (**HUM**), National Museum of Nature and Science, Tsukuba (**NSMT**), Sagamihara City Museum, Kanagawa, Japan (**SCM**), and private collections of Hiromu Kamezawa (Saitama-ken) (cKam). Type material of *Homoeusarufescens* is deposited in the Natural History Museum, London (**NHM**). Part of the paratypes from the Russian Far East are in the Institute of Biology and Soil Science, Vladivostok, Russia (**IBSS**).

The morphological observations and measurements were conducted using an Olympus SZX10. On the methods of dissection and preparation of permanent slides, we followed Maruyama (2004). Habitus photos were taken using a Canon 7D camera with a Canon MP-E 65 mm f/2.8 1–5X macro lens, and Neewer TT560/1Y strobe, and image stacking was conducted using Zerene Stacker ver. 1.04 (Zerene Systems LLC). Drawings were made using a microscope Olympus BX50 with an Olympus drawing tube attached.

Measurement definitions and abbreviations are shown as follows: BL, approximate body length; AL, antennal length; HW, head width; PL, pronotal length; PW, pronotal width; EL, elytral length (sutural length from apex of scutellum to posterior margin of elytra); EW, elytral width; HTL, hind tibial length. Measurements of dorsal morphology were made on 20 specimens of each species, and then the sexes were identified. Measurements of each segment of the antenna (six specimens of each species) were made on dissected specimens mounted in Euparal.

Symbiotic hosts were mostly identified by MM, F. Ito, and some by TN and K. Kinomura.

### ﻿Host ants

The scientific names of *Lasius* ants follow Bolton (1995), Radchenko (2005), and Boudinot et al. (2022). *Lasiusfuji* was formerly recognized as the eastern Palearctic population of *L.fuliginosus*. Recently, the Japanese population of “*Lasiusfuji*” has been suggested to be a species complex (Maruyama et al. 2013). Therefore, ‘Lasiuscf.fuliginosus’ is adopted as the name of the Japanese population here. *Lasiusfuliginosus* and allied species have long been classified in the subgenusDendrolasius. However, Boudinot et al. (2022) synonymized all the subgenera of *Lasius* with the genus *Lasius* and recognized five species groups, which is adopted here.

The species names of symbiotic hosts are abbreviated in “Type material” and “Additional material” as follows: *Lasiusfuliginosus* Group: *LFFJ*, *L.fuji* Radchenko, 2005; *LFFL*, *L.fuliginosus* (Latreille, 1798); *LF*cf*F*, Lasiuscf.fuliginosus; *LFM*, *Lasiusmorisitai* Yamauchi, 1979; *LFN*, *L.nipponensis* Forel, 1912; *LFO*, *L.orientalis* Karawajew, 1912; *LFS*, *L.spathepus* Wheeler, 1910; *LJ*, *Lasiusjaponicus* Santschi, 1941 (the *niger* group).

The distribution and host ant species of each species in the checklist is a synthesis of information from mostly Schülke and Smetana (2015), Maruyama et al. (2013), and this paper.

## ﻿Results

### ﻿Tribe Oxypodini Thomson, 1859

**Subtribe Dinardina Mulsant & Rey, 1873**

#### ﻿Genus Homoeusa

Taxon classificationAnimaliaColeopteraStaphylinidae

Kraatz, 1856

D74635A6-5F66-5D8F-8253-DAADCFA1AA1E

Homoeusa Kraatz, 1856: 76 (original description, type species: Euryusaacuminata Märkel, 1842, by monotypy); Kraatz 1857: 16 (diagnosis); Jacquelin du Val 1857: 11 (catalogue); Fenyes 1919: 389 (redescription); Bernhauer and Scheerpeltz 1926: 736 (synonymy, catalogue); Smetana 2004: 467 (catalogue); Schülke and Smetana 2015: 682 (catalogue).
Myrmobiota Casey, 1893: 594 (original description, type species: M.crassicornis Casey, 1893, by monotypy); Fenyes 1919: 392 (redescription); Bernhauer and Scheerpeltz 1926: 736 (catalogue; synonym of Homoeusa); Seevers 1978: 75 (redescribed).
Soliusa Casey, 1900: 53 (original description, type species: S.crinitula Casey, 1900, by monotypy); Fenyes 1919: 389 (synonym of Homoeusa); Bernhauer and Scheerpeltz 1926: 736 (catalogue; synonym of Homoeusa).

##### Diagnosis.

This genus is distinguished by the following combination of characteristics: body somewhat sub-limuloid; apex of ligula unilobed and round; antennae not or weekly clubbed; posterior margin of antennal insertion forming distinct latitudinal carina extending medially; spermatheca somewhat S-shaped.

##### Remarks.

As mentioned in Maruyama (2009), this genus is similar to *Losiusa* and *Aspidobactrus*, which are called the “*Homoeusa* genus complex”. Together with some other genera, these genera are classified in the subtribe Dinardina of the tribe Oxypodini (Schülke and Smetana 2015). The subtribe Dinardina is defined based on its limuloid body and shield-like pronotum (Seevers 1978). However, the monophyly of Dinardina (sensu Seevers 1978) has been rejected; the *Dinarda*+*Thiasophila* clade was distant from *Myrmobiota* (Osswald et al. 2013), which is a close relative of *Homoeusa* and often regarded as a junior synonym of *Homoeusa*. The *Homoeusa* genus complex is assumed to form a clade with *Myrmobiota* distant from *Dinarda*+*Thiasophila* given their symbiotic hosts and morphological characteristics of the head, ligula, and facial structure of the body. The relatedness of the *Homoeusa* genus complex and Nearctic genus *Decusa* Casey, 1900 was also suggested (see also Wasmann 1901). To reveal the relationships among these genera in detail, future phylogenetic research is necessary.

#### ﻿ Homoeusa rufescens

Taxon classificationAnimaliaColeopteraStaphylinidae

(Sharp, 1874), combination confirmed

5AC068EF-583D-5C96-9634-BFB233948F47

Japanese name: Hoso-hirata-ariyadori Figs 1–4 , 6–9 , 10–16 , 28 , 29

Thiasophila rufescens Sharp, 1874: 5; Fenyes 1919: 393 (catalogue); Bernhauer and Scheerpeltz 1926: 771 (catalogue); Smetana 2004: 488 (catalogue).
Homoeusa rufescens : Maruyama and Zerche 2014: 17 (mentioning actual generic affiliation); Schüelke and Smetana 2015; 682 (catalogue).

##### Type material.

**Figures 1–5.**
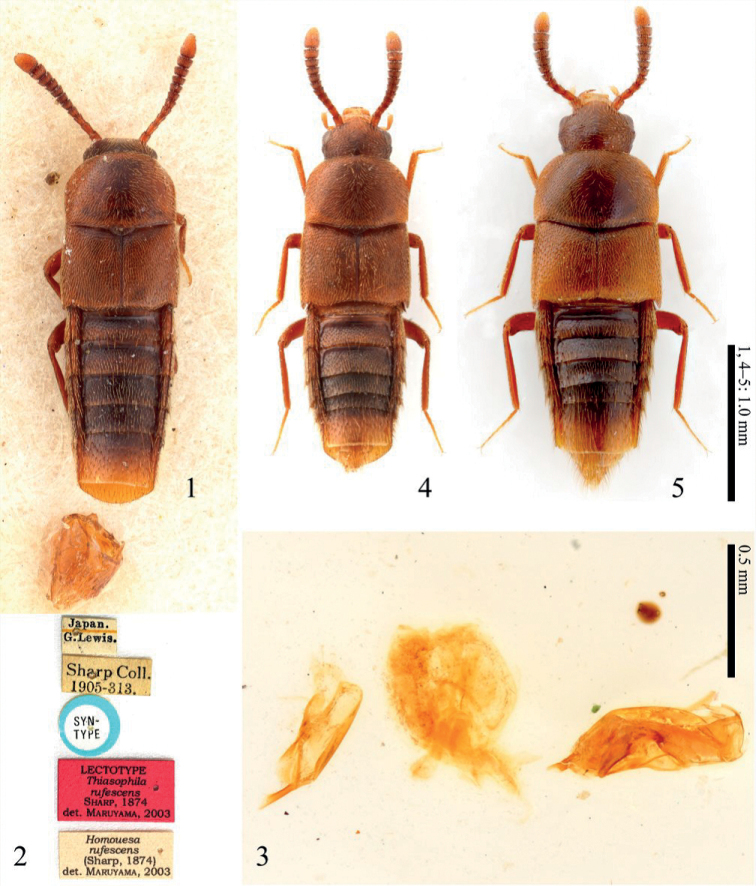
**1–3** Lectotype of *Homoeusarufescens* (Sharp, 1874): **1** habitus of the lectotype, male **2** labels of the lectotype **3** dissected parts of the lectotype **4** habitus of *Homoeusarufescens* from non-type specimens, female **5** habitus of the holotype of *Homoeusaovata* sp. nov., male.

***Lectotype*** (Figs 1–3), here designated, ♂, “Japan. Lewis.” / “Sharp Coll 1905-313” / “Syn-type” (blue round curator label) / “Lectotype *Thiasophilarufescens* det. Maruyama, 2003” (dissected by MM) (NHM). ***Paralectotypes***, 1 ♀, same data as lectotype but labelled “Type” (red round curator label) / “Thiasophilarufescens type D.S.” (NHM); 5 unsexed, same data as lectotype, but one is labelled “Nagasaki” (NHM).

##### Additional material.

**Japan: Honshû: Fukushima-ken**: 1 unsexed, Yukiwari-bashi, Nishigô-mura, 29. VII. 2000, T. Kobayashi. **Ibaraki-ken**: 2 unsexed, Inohana Pass, 29. V. 1994, Y. Hagino. **Tochigi-ken**: 1 unsexed, Tobiyama Castle, Utsunomiya-shi, 17–18. VI. 1998, M. Maruyama (*LFS*); 2 unsexed, 1♀, same locality, 17. VI. 1998, M. Maruyama (*LF*cf*F*); 2 unsexed, Shimokomoriya, Utsunomiya-shi, 6. VII. 1999, M. Maruyama; 3 unsexed, Sayado, Môka-shi, 15. VI. 2000, T. Kobayashi & H. Obata; 81 unsexed, Ichikai-machi, Haga-gun, 29. IV.–2. V. 2002, Seidai Nagashima. **Gunma-ken**: 27 unsexed, Mt. Sakurayama, Onishi-chô, 22. V. 1999, Shiho Arai (*LFS*); 2 unsexed, Sakurayama Park, Onishi-chô, 18. V. 1998, Shiho Arai (*LFS*); 26 unsexed, 7♂, 3♀, same locality, 9. V. 1998, Shiho Arai (*LJ*); 10 unsexed, Sakurayama, Onishi-chô, 9. V. 1998, Koji Toyoda (*LFS*); 5 unsexed, Nakanojo Forest Park, Nakanojô-machi, 8. VI. 2001, T. Watanabe. **Saitama-ken**: 15 unsexed, Shioyama, Ranzan-machi, 21. VI. 1996, Koji Toyoda; 10 unsexed, same locality, 8. VI. 1997, Koji Toyoda (*LF*cf*F*); 16 unsexed, same locality, 10. V. 1998, K. Toyoda (*LF*cf*F*); 3 unsexed, Shôgunsama, Ranzan-machi, 21. IV. 1999, K. Toyoda; 1 unsexed, Sugiyama, Ranzan-machi, 13. VII. 1998, K. Toyoda; 13 unsexed, Toki-gawa, Kamagata-mura, Ranzan-machi, 25. IV. 1999, K. Toyoda; 2 unsexed, Shiro-yama, Kamagata-mura, Ranzan-machi, 17. VI. 2000, K. Toyoda (*LF*cf*F*); 4 unsexed, Ranzan-keikoku, Kamagata-mura, Ranzan-machi, 14. V. 2000, K. Toyoda; 2 unsexed, Hashidake, Chichibu-shi, 26. IV. 1998, S. Arai; 2 unsexed, Nakano, Showa-machi, 4. V. 2002, Hiromu Kamezawa (*LFS*) (cKam); 1 unsexed, same locality, 26. VI. 2001, H. Kamezawa (*LF*cf*F*) (cKam); 2 unsexed, same locality, 4. V. 2002, H. Kamezawa (*LF*cf*F*) (cKam); 5 unsexed, same locality, 4. V. 2003, H. Kamezawa (*LF*cf*F*) (cKam); 1 unsexed, Yokoze-machi, Chichibu-shi, 10–11. V. 1995, Yoshinori Kaneko, Akio Ito, Satoshi Tsuboyama. **Chiba-ken**: 1 unsexed, Mt. Kiyosumi, Amatsukominato, 9. VI. 1991, T. Takeda; 1 unsexed, Azeta, Sakura-shi, 20. VI. 1998, M. Maruyama (*LFS*); 1 unsexed, 1♀, same locality, 23–24. VI. 1998, M. Maruyama (*LFS*); 2 unsexed, same locality, 26. VI. 1998, M. Maruyama (*LFS*). **Tôkyô-to**: 1 unsexed, Mt. Takao, 1. V. 1985, S. Nomura; 2 unsexed, same locality, 13. V. 1985, S. Nomura; 2 unsexed, Takao-san (450 m in alt.), Hachioji-shi, 4. VI. 2001, M. Maruyama (*LFS*); 1 unsexed, Otomeyama Park, Shinjuku-ku, 13. V. 1985, S. Kubota; 3 unsexed, Dokan-bori, Imperial Palace, 17. V. 2000, T. Shimada (NSMT); 34 unsexed, Kami-dokan-bori, Imperial Palace, 17. V. 2000, Shiho Arai (*LF*cf*F*) (MSMT). **Kanagawa-ken**: 2 unsexed, Kawasaki-shi, 1. V. 1985, S. Kubota; 3 unsexed, Ikuta-Ryokuchi, Kawasaki-shi, 13. IV. 2002, K. Matsumoto; 1 unsexed, Mt. Masukata, Kawasaki-shi, 15. VI. 1996, K. Kawada; 1 unsexed, Jinmu-ji, Zushi-shi, 20. VI. 2003, M. Maruyama; 1 unsexed, Mt. Tanzawa, 26. VI. 1983, Y. Hirano; 1 unsexed, Kawana, Fujisawa, 19. VI. 2000, T. Watanabe (*LFS*); 1 unsexed, same locality, 7. V. 2001, T. Watanabe (*LF*cf*F*); 3 unsexed, 1♂, same locality, 14. V. 2001, T. Watanabe; 6 unsexed, Toya, Tsukui, 11. V. 1976, Ryo Kiryu (SCM); 6 unsexed, same locality, 29. IV. 1977, Ryo Kiryu (SCM); 2 unsexed, Mikage, Tsukui, 18. V. 1976, Ryo Kiryu (SCM); 1 unsexed, same locality, 23. IV. 1977, Ryo Kiryu (SCM); 1 unsexed, same locality, 9. VI. 1979, Ryo Kiryu (SCM). **Yamanashi-ken**: 2 unsexed, Karumizu-rindô, (1400 m in alt.), Narusawa, 29. VI. 2011, T. Watanabe. **Shimane-ken**: 1 unsexed, Urahikimi, 6. VI. 1988, S. Nomura. **Okayama-ken**: 1 unsexed, Ono Shine, Kawakami, Ohara-chô, Mimasaka-shi, 7. VI. 2009, Yoshifumi Fuzitani; 5 unsexed, same locality, 5. V. 2009, Yoshifumi Fuzitani (*LFS*). **Yamaguchi-ken**: 3 unsexed, Nishimagura, (100 m in alt.), Kusunoki, 30. V.–1. VI. 2000, Toshio Kishimoto. **Shikoku: Kagawa-ken**: 7 unsexed, 1♂, Usa-Jinja, Nagaona, Sanuki-shi, 31. V. 2001, M. Maruyama (*LFS*); 11 unsexed, 1♂, 1♀, Ôtaki-san, Shionoe-chô, 2. VI. 2001, M. Maruyama (*LFN*); 1 unsexed, Fujio-Jinja, Nishiuta-chô, Takamatsu-shi, 31. V. 2001, M. Maruyama (*LFS*); 4 unsexed, same locality, 1. VI. 2001, M. Maruyama (*LFN*); 5 unsexed, Furodani, Miki-chô, 30. VII. 2000, K. Izawa; 1 unsexed, Kamiyama, Miki-chô, 1. VI. 2001, M. Maruyama (*LFS*); 4 unsexed, Ôtawa, Nagano-chô, 22. V. 2000, F. Ito; 11 unsexed, Atago-yama, Kotohira-chô, 1. VI. 2001, M. Maruyama (*LFS*). **Ehime-ken**: 1♂, Sugitate, 17. VI. 2017, Yu Hisasue. **Kyûshû: Fukuoka-ken**: 1 unsexed, Hikosan, Biol. Lab. KU, Soeda-machi, 8–10. V. 1957, K. Morimoto; 1 unsexed, same locality, 7. VI. 1993, S. Nomura; 3 unsexed, Hikosan, Biol. Lab. KU (750 m in alt.), Soeda-machi, 22. V. 2011, M. Maruyama (*LF*cf*F*); 1 unsexed, Kusaba, Nishi-ku, Fukuoka-shi, 28. IV. 2018, Tsubasa Nozaki; 1 unsexed, Motooka, Nishi-ku, Fukuoka-shi, 29. IV. 2020, Tsubasa Nozaki; 1 unsexed, Mt. Shioji, Otogana, Onojo-shi [33.5416°N, 130.5023°E], 28. V. 2017, Yu Hisasue (*LFS*). **Saga-ken**: 1 unsexed, Tôsen-zan, Ureshino-shi, 29. IV. 2018, Mitsuyasu Nishida (fit). **Nagasaki-ken**: 4 unsexed, Tanukinoo, Masuragahara-machi, Ômura-shi, 5. V. 2018, Mitsuyasu Nishida. **Kumamoto-ken**: 1 unsexed, Shiraga-dake, 4. XI. 1984, M. Ohara (HUM).

##### Diagnosis.

It is distinguished from the other species of the genus by the following combination of characteristics: body small, slender and subparallel-sided; pronotum less transverse (PW/PL, 1.38–1.47), widest near middle, posterolateral angle obtuse, posterior margin hardly sinuate; apical lobe of male aedeagus thick with round sheet-like projection; apical lobe of paramere straight; velum broad and round.

##### Redescription.

***Body*** (Figs 1, 4) small, very slender, subparallel-sided; dorsal surface mostly slightly polished.

**Figures 6–9.**
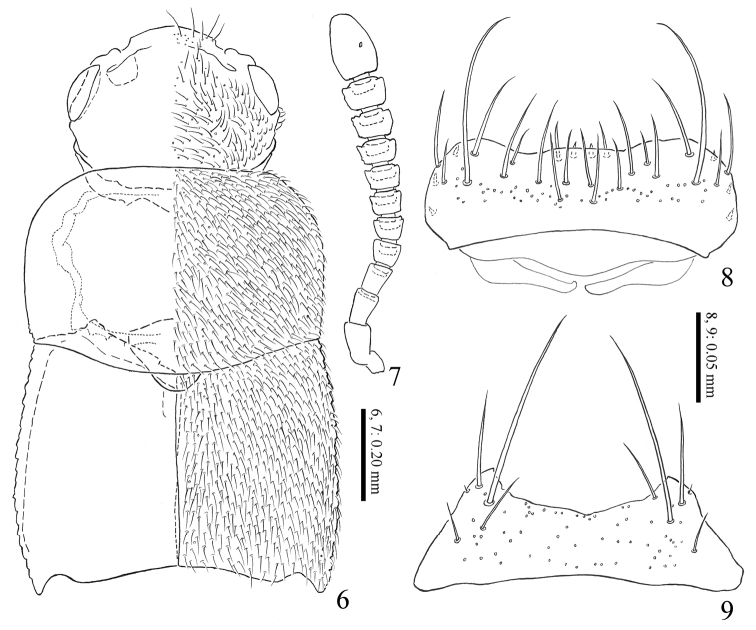
*Homoeusarufescens* Sharp, 1874 **6** fore body **7** right antenna **8** labrum **9** mentum.

**Figures 10–16.**
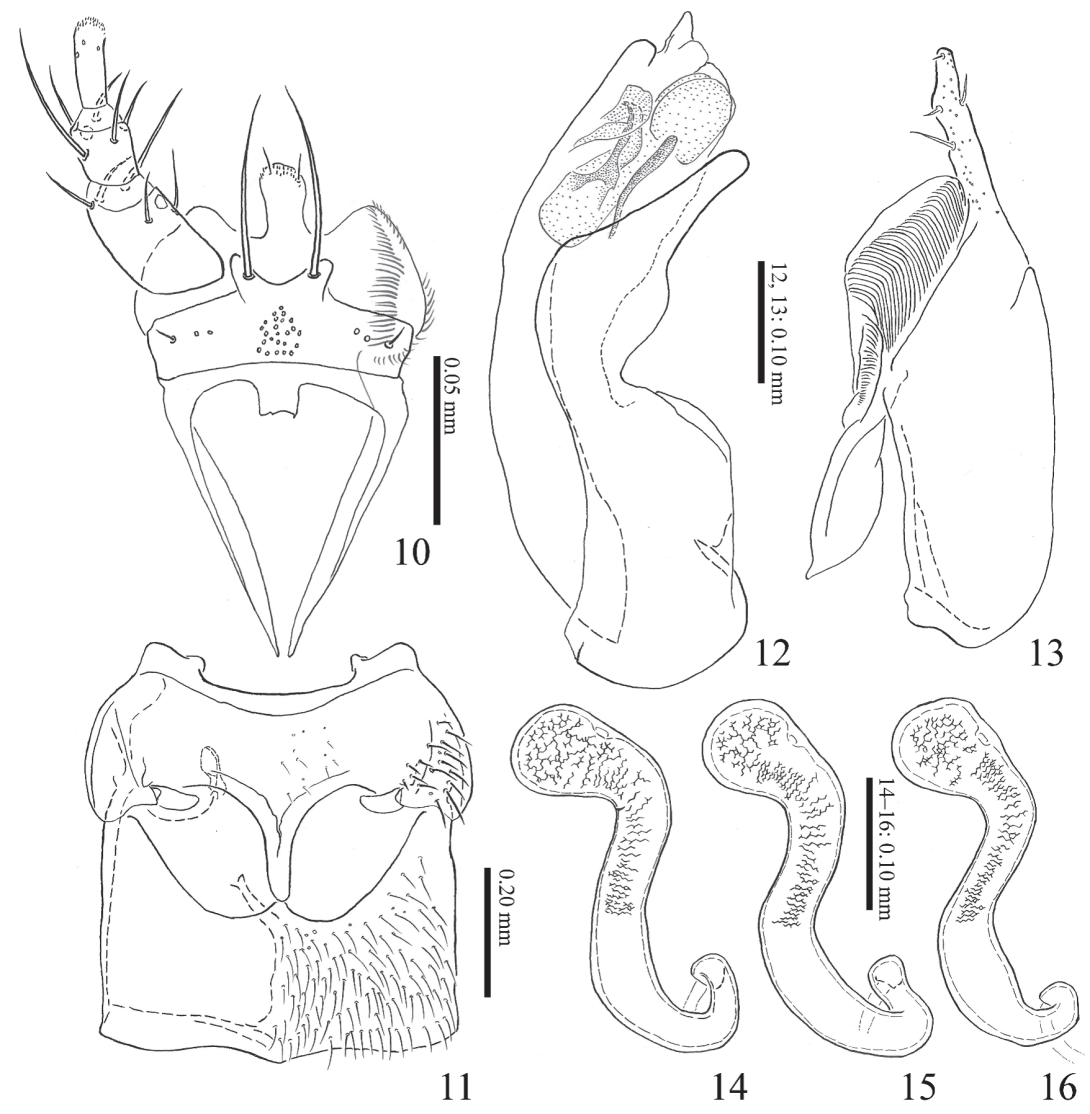
*Homoeusarufescens* Sharp, 1874 **10** labium **11** meso-, metaventrite **12** median lobe of aedeagus from lateral view **13** paramere of aedeagus from lateral view **14–16** spermatheca.

***Head*** (Fig. 6) relatively large, reddish brown; frontal margin with few long setae; carina of antennal insertion gently sinuate; eye large. Antennae (Fig. 7) stout, as long as head and pronotum combined, reddish brown, but segments I–IV and XI paler; segment I dilated, widest near apex; segment II slightly longer than III, widened apically; segment III widened apically; segment IV–X widened apically, each apical margin fringed with small teeth and longer than wide; segments IV as long as wide; segments V–X slightly wider than long; segment XI oblong oval. Labrum (Fig. 8), apical margin concave, surface with 20 setae; epipharynx with 2 pairs of sensillae on anterior margin, and 3 pairs of micro setae on lateral margin. Mentum (Fig. 9) with 3 pairs of setae and 1 pair of microsetae; anterior margin deeply concave. Prementum (Fig. 10) with many pseudopores, and with 2–3 real pores and 1 setal pore on each side near middle lateral margin. Ligula elongate, apical margin with 1 pair of spinule and several sensilla. Labial palpus, segment II and III each with 5 setae.

***Pronotum*** (Fig. 6) convex, subrectangular, less transverse, widest near middle; posterolateral angle obtuse, posterior margin hardly sinuate, brownish red to brownish black; surface finely covered with setae and punctures. Elytra (Fig. 6) slightly widened posteriorly, posterior margins shallowly notched near lateral corners, brownish red; surface finely covered with setae and punctures, moderately reticulated. Mesoventral processes (Fig. 11) narrow, with medial carina forming Y-shaped, apex rounded; mesocoxal cavities separated; metaventral process weakly produced.

***Abdomen*** elongate, slightly narrowed posteriad; surface sparsely covered with short setae and each posterior margin with long stout setae; polished and weekly reticulated.

**Male**: 8^th^ sternite longer than wide, weakly rounded apically. Median lobe of aedeagus (Fig. 12), apical lobe of aedeagus thick with round flattened projection on ventral side; apical lobe of paramere (Fig. 13) short and straight; velum broad and round; ventral margin of paramerite almost straight.

**Female**: 8^th^ sternite longer than wide, weakly rounded apically. Spermatheca (Figs 14–16), apical part moderately swollen; apical 3/5 with inner wall densely reticulated.

##### Measurements.

Body shape (*N* = 20): BL ≈ 1.6–2.7; AL, 0.64–0.75; HW, 0.41–0.47; PL, 0.44–0.50; PW, 0.61–0.70; EL, 0.38–0.44; EW, 0.65–0.73; HTL, 0.42–0.48; PW/PL, 1.38–1.49; PW/HW, 1.38–1.57; AL/HW, 1.45–1.77. Aspect ratio (length/width) of each antennal segment from I to XI (*N* = 6): 1.36–1.58, 1.43–1.71, 1.15–1.22, 0.63–0.77, 0.52–0.63, 0.43–0.53, 0.44–0.50, 0.39–0.49, 0.43–0.51, 0.49–0.56, 1.26–1.54.

##### Variation.

In most individuals, the pronotum tends to be brownish red, but the color varies from brownish red to brownish black.

##### Distribution.

Japan (Honshû, Shikoku, Kyûshû).

##### Bionomics.

From April to July, this species can be found in the trails of the *Lasiusfuliginosus* species group (Fig. 28). When they encounter host ants, they pause briefly until the ants ignore them and then start walking. TN observed that they ate prey that the ants were trying to carry but dropped. This species is sometimes observed to climb on food that the host ant is carrying and eat it (Fig. 29).

##### Symbiotic hosts.

Lasiuscf.fuliginosus, *L.morisitai*, *L.nipponensis*, *L.spathepus*.

##### Remarks.

The original description suggested that the syntypes included another species (Sharp 1874). Based on our examination of the syntype, it includes two species, as mentioned in the original description. Here, the specimens of the more slender species that more closely match the original description are designated as the lectotype and paralectotype. The specimens that were judged a different species were excluded from the syntypes of *H.rufescens* and included in the paratypes of the following species. As is often the case with old specimens, the proteins in the lectotype were denatured and potassium hydroxide did not sufficiently dissolve the muscle of the genital organs (Fig. 3), but the morphological characteristics of this species were fully observed.

In the original description, “*Thiasophilarufescens*” is “found with *Formicajaponica*” (Sharp 1874). Ruzsky (1912) established the subgenus ‘*Dendrolasius*’ (=*Lasiusfuliginosus* species group), with “*Formicafuliginosa*” as the type species. There were no *Homoeusarufescens* specimens collected from *Formica* ants in the present study, so the syntypes were also likely collected from the *Lasiusfuliginosus* species group. A single specimen was pinned with a *Lasiusjaponicus* (*niger* group), but this is considered to be a coincidence.

#### ﻿ Homoeusa ovata

Taxon classificationAnimaliaColeopteraStaphylinidae

Nozaki & Maruyama sp. nov.

9D6FA575-3250-528A-95AE-1B050E7BB766

https://zoobank.org/6638F337-2AAC-419F-9334-01EA46D98C90

Japanese name: Hime-hirata-ariyadori Figs 5 , 17–20 , 21–27 , 30 , 31

##### Type material.

***Holotype*.** “Mt. Maruyama / Sapporo-shi / <Hokkaido, JAPAN> / 6. VI. 1988 / M. Maruyama leg. / trail of ants” (*LF*cf*F*). ***Paratypes*.** 1♂, (ex type series of “*Thiasophilarufescens*”), “Japan. Lewis.” / “Sharp Coll 1905-313” (no other data) (NHM). Paratypes. 1 unsexed (ex type series of “*Thiasophilarufescens*”), same data but labelled “rufescens var.?” (NHM). **Russia: Primorskyi krai**: 1 unsexed, Kamenushka, Ussuryisk (MMLASRUS07), 28. V. 2005, M. Maruyama (*LFO*); 1 unsexed, same locality (MMLASRUS09), 28. V. 2005, M. Maruyama (*LFO*) (IBSS); 1 unsexed, 1♂, same locality (MMLASRUS11), 28. V. 2005, M. Maruyama (*LFN*); 1 unsexed, Bukhta Vityaz, Poluostrov Gamov, Khasanskyi (MMLASRUS14), 29. V. 2005, M. Maruyama (*LFO*); 2 unsexed, same locality (MMLASRUS17), 31. V. 2005, M. Maruyama (*LFO*) (IBSS); 2 unsexed, same locality (MMLASRUS19), 31. V. 2005, M. Maruyama (*LFFJ*); 6 unsexed, 2♀, Okeanskaya, Vladivostok (MMLASRUS23), 1. VI. 2005, M. Maruyama (*LFO*); 6 unsexed, 1♂, 1♀, Nadezhdinskyi, Khasanskyi, 1. VI. 2005, M. Maruyama (*LFFJ*) (IBSS); 2 unsexed, 3♂, Arisimovka, 70 km E Vladivostok, [43.11°N, 132.41°E], 5. VI. 1993, L. Zerche (*LFO*); 2 unsexed, Sibir or. Ussuri Vladivostok, 1919, Dr. Jureček. **Korea**: 8 unsexed, 1♂, Janggoksa, Chilgab-san, Cheongyang-gun, Chungnam, 23. VI. 2000, Shuhei Nomura (*LFS*); 70 unsexed, 1♂, Jeonglyeong Chi Samnae Myeon Cheonla-buk Do, 12. VII. 1991, S. Nomura (*LFO*); 18 unsexed, 3♂, 1♀, same locality, 12. VII. 1991, S. Nomura. **Japan: Hokkaido**: 23 unsexed, Hyakumatsu-zawa, Sapporo-shi, 8. VI. 1998, M. Maruyama (*LF*cf*F*); 50 unsexed, Hakken-zan, Sapporo-shi, 31. V. 2002, M. Maruyama (*LF*cf*F*); 54 unsexed, same locality, 31. VI. 2002, M. Maruyama (*LF*cf*F*); 17 unsexed, 1♀, same locality, 1. VI. 2002, M. Maruyama (*LFO*); 92 unsexed, 1♂, 1♀, same locality, 1. VI. 2002, M. Maruyama (*LFN*); 267 unsexed, 2♂, 2♀, same locality, 1. VI. 2002, M. Maruyama (*LF*cf*F*); 2 unsexed, Kannon-zawa, Sapporo-shi, 1. V. 2002, M. Maruyama; 6 unsexed, same locality, 20. V. 2002, M. Maruyama; 27 unsexed, same locality, 31. V. 2002, M. Maruyama (*LF*cf*F*); 4 unsexed, 1♂, 5♀, same locality, 1 VI 2002, M. Maruyama (*LF*cf*F*); 23 unsexed, Maruyama, Sapporo-shi, 6. VI. 1998, M. Maruyama (*LF*cf*F*) (same data as holotype); 3 unsexed, Hitsujigaoka, Sapporo-shi, 18. V. 2000, M. Maruyama (*LFS*); 2 unsexed, Nopporo, 17. VII. 1990, M. Ohara (HUM); 12 unsexed, Nopporo, Ebetsu-shi, 13. VI. 1999, M. Maruyama (*LF*cf*F*); 20 unsexed, 2♀, Ôsawaguchi, Nopporo-shinrin-kôen, Ebetsu-shi, 16–19. VI. 2001, S. Hori; 46 unsexed, same locality, 16–19. VI. 2001, S. Hori (*LFO*); 25 unsexed, same locality, 2. VI. 1999, M. Maruyama; 2 unsexed, Tomambetsu, Nopporo, Ebetsu-shi, 29. V. 2002, M. Maruyama (*LF*cf*F*); 1 unsexed, Nozaki, Bihoro-chô, 23. VI. 2001, Y. Kida; 1 unsexed, Taihei, Maruseppu-chô, 29–31. V. 2000, Y. Kida; 104 unsexed, 1♀, same locality, 16–17. VI. 2000, Y. Kida; 4 unsexed, same locality, 11–12. VIII. 2000, Y. Kida (*LF*cf*F*); 36 unsexed, same locality, 19–21. VIII. 2000, Y. Kida (*LF*cf*F*); 1 unsexed, 2♂, 1♀, same locality, 25. VIII. 2000, M. Maruyama (*LF*cf*F*); 1 unsexed, Makoi, Shari-chô, 27. V. 2000, Y. Kida; 2 unsexed, same locality, 28. V. 2000, Y. Kida; 53 unsexed, 1♂, same locality, 24. VI. 2002, Y. Kida; 4 unsexed, same locality, 13. VI. 2002, Y. Kida; 44 unsexed, Kanayama, same locality, 18–24. VI. 2002, Yasunari Kida; 7 unsexed, Midorioka-kôen, Kitami-shi, 17. VI. 2001, M. Maruyama; 3 unsexed, Hebinuma, Teshio-gawa, Teshio-chô, 9. VII. 1992, S. Hori; 2 unsexed, Kamishunbetu, 20. VII. 1977, Naomi; 2 unsexed, Yûtoku, Ôtaki-mura, 24. VIII. 2001, M. Maruyama; 4 unsexed, Mukôengaru Engaru-chô, 25. V. 2000, Y. Kida (*LF*cf*F*); 2 unsexed, Miwa, Koshimizu-chô, 28. VII. 2001, S. Kuwahara; 1 unsexed, Mt. Kariba, Shiribeshi, 13. VI. 1986, S. Nomura; 1 unsexed, Himenuma, Rishiri Is, 4. IX. 1990, T. Kishimoto. **Honshû: Aomori-ken**: 1 unsexed, Hirosaki, 3. VII. 1960, Y. Murakami; 2 unsexed, same locality, 5. VII. 1960, Y. Murakami. **Miyagi-ken**: 1 unsexed, Aoba-yama, Sendai-shi, 17. VII. 2004, K. Mizota; 6 unsexed, Naruko-onsen, Naruko-chô, 14–17. VI. 1999, M. Sano (*LFO*). **Akita-ken**: 7 unsexed, 1♀, Tamagawa, Tazawako-machi, 12–13. VI. 1999, M. Sano (*LF*cf*F*). **Fukushima-ken**: 6 unsexed, Mizuhiki, Toteiwa-mura, 12. VI. 2004, H. Kamezawa (cKam); 33 unsexed, 2♂, Kashi Spa., Nishigo-mura, 16. VI. 1998, M. Maruyama (*LF*cf*F*); 12 unsexed, same locality, 17. VI. 1998, M. Maruyama (*LF*cf*F*); 1 unsexed, Nanairi, Hinoemata, 25. VII. 1996, S. Naomi. **Ibaraki-ken**: 2 unsexed, Inohana Pass, 29. V. 1994, Y. Hagino. **Tochigi-ken**: 1 unsexed, Yumoto, Nikkô, 29. IV. 1982, S. Naomi; 1 unsexed, Ichikai-machi, Haga-gun, 29. IV.–2. V. 2002, Seidai Nagashima. **Gunma-ken**: 5 unsexed, Sakurayama, Onishi-chô, 9. V. 1998, Koji Toyoda (*LFS*); 5 unsexed, same locality, 9. V. 1998, Shiho Arai (*LFS*); 2 unsexed, same locality, 9. V. 1998, Shiho Arai (*LJ*); 1 unsexed, same locality, 22. V. 1999, Shiho Arai (*LFS*); 10 unsexed, Nageishi-tôge, Fujioka-shi, 5. VI. 2001, T. Watanabe; 8 unsexed, Nakanojô-forest-park, Nakanojô-machi, Agatsuma-gun, 8. VI. 2001, T. Watanabe. **Saitama-ken**: 3 unsexed, Shiro-yama, Kamagata, Ranazan-machi, 17. VI. 2000, K. Toyoda; 1 unsexed, 1♂, Ranzan-keikoku, Kamagata-mura, Ranzan-machi, 14. IV. 2000, K. Toyoda; 1 unsexed, same locality, 25. IV. 2000, K. Toyoda; 1 unsexed, same locality, 21. IV. 1999, K. Toyoda; 4 unsexed, Shioyama, Ranzan-machi, 8. V. 1998, K. Toyoda; 7 unsexed, same locality, 10. V. 1998, K. Toyoda; 11 unsexed, same locality, 10. V. 1998, K. Toyoda (*LF*cf*F*); 3 unsexed, 1♂, Toki-gawa, Kamagata-mura, Ranzan-machi, 25. IV. 1999, K. Toyoda; 5 unsexed, Shiroishi-toge, Higashichichibu-mura, 20. VI. 1999, Shiho Arai; 1 unsexed, same locality, 17. VI. 2000, K. Toyoda (*LFS*); 4 unsexed, Nr. Shiroishi Pass Higashichichibu-mura, 27. VI. 1998, K. Toyoda; 1 unsexed, Chichibu-kôgen, Higashichichibu-mura, 3. VII. 1999, K. Toyoda; 1 unsexed, same locality, 3. VII. 1999, K. Toyoda; 3 unsexed, Hashidake, Chichibu-shi, 17. VII. 1999, Shiho Arai (*LJ*); 1 unsexed, same locality, 26. IV. 1998, K. Toyoda (*LFS*); 1 unsexed, Koakazawa, (1,000 m in alt), Iri Kawa, Mt.Hakutai san, Ootaki-chiku, Chichibu-shi, 7. VI. 2002, Koji Toyoda; 1 unsexed, Hashidate Riv., Chichibu-shi, 7. VI. 2002, Koji Toyoda; 3 unsexed, Mt. Jyouminesan Minao-chô, 29. V. 1999, Shiho Arai (*LF*cf*F*); 1 unsexed, Nr. Ohno Pass Takigawa-mura, 5. VIII. 1998, K. Toyoda (*LFS*). **Chiba-ken**: 2 unsexed, Matuzaki, Inzai-shi, 3. V. 1991, T. Takeda. **Tôkyô-to**: 1 unsexed, Mt. Takao, 1. V. 1985, S. Kubota; 35 unsexed, 3♂, 2♀, Takao-san (450 m in alt.), Hachiôji-shi, 4. VI. 2001, M. Maruyama (*LFS*); 4 unsexed, Takao-san, Hachiôji-shi, 1. VII. 1998, M. Maruyama; 2 unsexed, same locality, 4. VII. 1998, M. Maruyama; 1 unsexed, same locality, 4. VII. 1998, M. Maruyama (*LFS*); 1 unsexed, Oyama, Machida-shi, 19. V. 1991, T. Kishimoto. **Kanagawa-ken**: 6 unsexed, Kawana, Fujisawa-shi, 14. V. 2001, T. Watanabe (*LFS*); 4 unsexed, Jimmu-ji, Zushi-shi, 20. VI. 2003, M. Maruyama; 1 unsexed, Toya, Tsukui, 29. IV. 1977, Ryo Kiryu (SCM); 1 unsexed, same locality, 14. V. 1976, Ryo Kiryu (SCM); 1 unsexed, Nanzawa, Tsukui, 14. V. 1977, Ryo Kiryu (SCM); 1 unsexed, Hasuge-san, Aikô, 3. V. 1963, Ryo Kiryu (SCM); 1 unsexed, Minami-Ashigara, 11. II. 1975, Y. Hirano; 2 unsexed, Fudakake, Higashi-Tanzawa, 16. VI. 1984, Y. Hirano; 1 unsexed, Mt. Tanzawa, 26. II. 1973, Y. Hirano; 1 unsexed, Kawasaki, 1. V. 1985, 1. V. 1985; 1 unsexed, Kozuka-yama, Hakozaki-chô, 25–26. V. 1995, Yoshinori Kaneko, Akio Ito, Satoshi Tsuboyama; 6 unsexed, 1♂, Niiharu-shiminno-mori, Yokohama-shi, 21. V. 2001, T. Watanabe (*LFO*). **Niigata-ken**: 2 unsexed, Yuzawa-machi, Minami-uonuma-gun, 20–22. VI. 2002, S. Nagashima (*LFS*); 2 unsexed, Tairai, 3. VII. 1985, S. Nomura. **Ishikawa-ken**: 2 unsexed, Ichinose pass Shiramine-Village, 25. V–7. VI.2003, Katsuyuki Nakata (fit). **Yamanashi-ken**: 8 unsexed, Hikawarindô, Daibosatsu-rei, Koushû-shi, 24. VI. 2001, T. Watanabe; 4 unsexed, Ashiyu-onsen, Ashiyu-mura, 23. V. 2001, T. Watanabe (*LF*cf*F*); 1 unsexed, same locality, 24. V. 2001, T. Watanabe; 3 unsexed, Shiroidaira Dôshi-mura, 17.VII.2001, T. Watanabe; 4 unsexed, Gozaishi-kôsen (900 m in alt.), Nirasaki-shi, 25. VII. 2002, S. Nomura (*LFN*); 2 unsexed, Karumizu-rindô, (1400 m in alt.), Narusawa, 29. VI. 2011, T. Watanabe. **Nagano-ken**: 1 unsexed, Kakuma Valley, Sanada-machi, 17. VI. 2001, T. Watanabe; 1 unsexed, Midarebashi, Honjô-mura, 21. VII. 1999, T. Watanabe; 52 unsexed, Penshionmura, Hara-mura, 11. VII. 1999, Shiho Arai (*LFO*); 2 unsexed, Otari-onsen (1100 m in alt.), Otari-mura, Kita-azumi-gun, 16. V. 2004, H. Kamezawa (cKam); 2 unsexed, Fujii, Satoyamabe, Matsumoto-shi, 9. VI. 2004, T. Komatsu; 1 unsexed, same locality, 11. VI. 2004, T. Komatsu; 1 unsexed, same locality, 21. VI. 2004, T. Komatsu. **Gifu-ken**: 6 unsexed, Ogamigo (800 m in alt.) Shôkawa-mura, 5–6. VIII. 1998, M. Maruyama (*LFS*); 4 unsexed, Mt. Kinkazan, Gifu-shi, 11. VI. 2003, K. Kinomura (*LFS*); 1 unsexed, Isshiki, Shôkawa-chô, Takayama-shi, 1. VIII. 2004, K. Kinomura (*LFN*); 51 unsexed, Isshiki, Shôkawa-chô, Takayama-shi, 8. VI. 2013, K. Kinomura (*LFM*); 29 unsexed, Nabedaira-kôgen, Kansaka, Okuhida-onsenkyô, Takayama-shi, 30. VI. 2013, K. Kinomura (*LFN*); 20 unsexed, Nabedaira-kôgen, (1305 m in alt.) Kansaka, Okuhida-onsenkyô, Takayama-shi, (1305 m in alt.), 30. VI. 2013, K. Kinomura (*LFN*). **Wakayama-ken**: 1 unsexed, Mt. Gomandan, 22–23. VI. 1981, S. Naomi. **Tottori-ken**: 1♂, Mt. Daisen, 3–5. VI. 1980, S. Naomi; 3 unsexed, same locality, 10. VI. 1986, S. Nomura (*LFS*); 10 unsexed, 1♂, Urahikimi, Shimane-ken, 6. VI. 1988, S. Nomura (*LFS*). **Okayama-ken**: 2 unsexed, Ono Shine, Kawakami, Ohara-chô, Mimasaka-shi, 5. V. 2009, Yoshifumi Fujitani; 1 unsexed, same locality, 24. V. 2009, Yoshifumi Fujitani; 1 unsexed, same locality, 7. VI. 2009, Yoshifumi Fujitani. **Hiroshima-ken**: 11 unsexed, Nakatsuya, Hatsukaichi-shi, 7. VI. 1987, S. Nomura; 1 unsexed, Mt. Garyu, 27. VI. 1987, S. Nomura (*LF*cf*F*). **Yamaguchi-ken**: 10 unsexed, Nishimagura, (100 m in alt.), Kusunoki, 30. V. – 1. VI. 2000, Toshio Kishimoto; 3 unsexed, Momijidani park, Yokoyama, Iwakuni-shi, 28. V. 2011, M. Shimono (*LFS*). **Shikoku: Kagawa-ken**: 5 unsexed, Furodani, Miki-chô, 30. VII. 2000, K. Izawa (*LF*cf*F*); 108 unsexed, 3♂, 1♀, Ôtaki-san, Shionoe-chô, 2. VI. 2001, M. Maruyama et al. (*LFN*); 21 unsexed, Atago-yama, Kotohira-chô, 1. VI. 2001, M. Maruyama et al. (*LFS*); 22 unsexed, Fujio-jinja, Nishi-ueta-chô, Takamatsu-shi, 31. V. 2001, M. Maruyama et al. (*LFN*); 2 unsexed, same locality, 1. VI. 2001, M. Maruyama et al. (*LFN*); 22 unsexed, Daisen-zan, Kotonami-chô, 1. VI. 2001, M. Maruyama et al. (*LFM*); 13 unsexed, Usa-jinja, Nagaona, Sanuki-shi, 31. V. 2001, M. Maruyama et al. (*LFS*); 3 unsexed, Shirahige-jinja, Ayakami-chô, 25. IV. 2004, H. Fujimoto (*LFM*). **Ehime-ken**: 1♂, Sugitate, 17. VI. 2017, Yu Hisasue; 2 unsexed, Waki-ga-fuchi Park (120–220 m in alt.), Sue-machi, Matsuyama-shi [33.87°N, 132.83°E], 4. V. 2017, Yu Hisasue (Tullgren); 1 unsexed, Mt. Ishizuchi (1400 m in alt.), Saijô-shi, 29. VI. 2014, Yu Hisasue; 1 unsexed, Minamikume-machi, Matsuyama-shi [33.8333°N, 132.8184°E], 25. IV. 2015, Yu Hisasue (Tullgren). **Kyûshû: Fukuoka-ken**: 1 unsexed, Hakozaki, Fukuoka-shi, 12. V. 1979, K. Yamagishi; 2 unsexed, 1♂, Hikosan, Biol. Lab. KU (750 m in alt.), Soeda-machi, 22. V. 2011, M. Maruyama (*LF*cf*F*). **Saga-ken**: 2 unsexed, Mt. Mifume, 15. V. 2019, S. Nomura; 2 unsexed, Mt. Kurokami-yama (400 m in alt.), Takeo-shi [33.2130°N, 129.9044°E], 9. V. 2021, Yu Hisasue (*LF*cf*F*); 4 unsexed, 1♂, Kurokami-yama, Arita-chô, 15. V. 2019, Mitsuyasu Nishida. **Nagasaki-ken**: 3 unsexed, 1♂, Tanukinoo, Masuragahara-machi, Ômura-shi, 5. V. 2018, Mitsuyasu Nishida; 1 unsexed, Todoroki Valley, 1. VI. 1987, S. Naomi; 1♂, Suwa Shine, Nagasaki-shi, 2. V. 1985, S. Nomura; 1 unsexed, Tomikawa-keikoku, Isahaya-shi, 28. IV.–1. V. 2008, T. Iwai.

##### Diagnosis.

It is distinguished from the other species of the genus by the following combination of characteristics: body subparallel-sided; pronotum convex and strongly transverse (PW/PL, 1.54–1.68), widest near middle, sides barely protrude, posterolateral angle obtuse and posterior margin hardly sinuate; apical lobe of male aedeagus S-shaped with small lanceolate sheet-like projection; apical lobe of paramere (Fig. 24) curved ventrally and thickened apicad with four setae gathering to apex; velum emarginate at middle. This species is especially similar to *H.rufescens* and slightly resembles *H.japonica* and *H.prolongata* in habitus. However, this species is easily separated from the three species by the shape of aedeagus and dorsal characteristics: *H.rufescens* by pronotum aspect ratio (less transverse in *H.rufescens*); *H.japonica* by body size and pronotum shape (posterior margin is moderately sinuate in *H.japonica*); and *H.prolongata* by pronotum shape (more flattened and hemicircular, posterolateral angle gently acute in *H.prolongata*).

##### Description.

***Body*** (Fig. 5) small, slender, subparallel-sided; dorsal surface mostly moderately polished.

**Figures 17–20.**
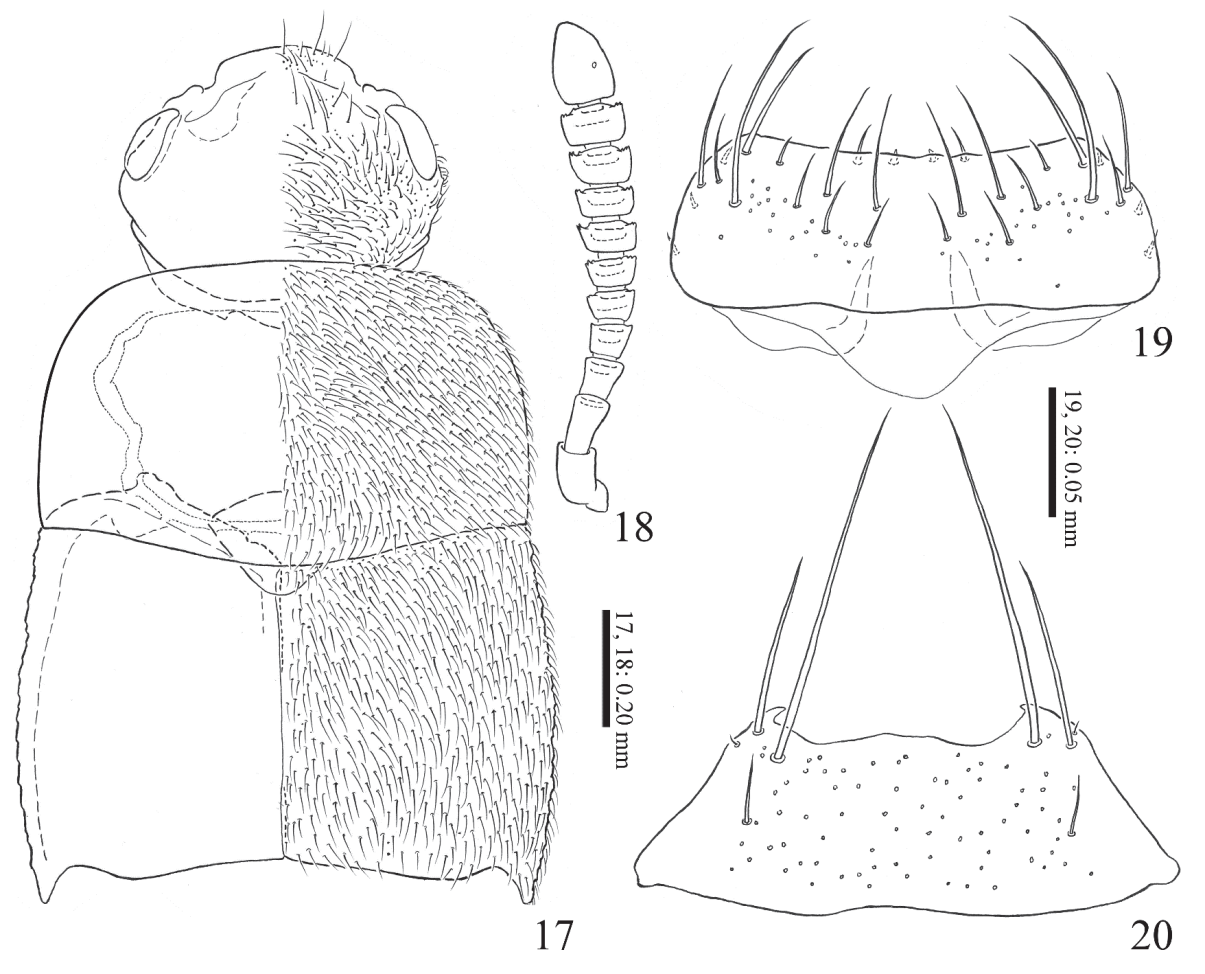
*Homoeusaovata* sp. nov. **17** fore body **18** right antenna **19** labrum **20** mentum.

**Figures 21–27.**
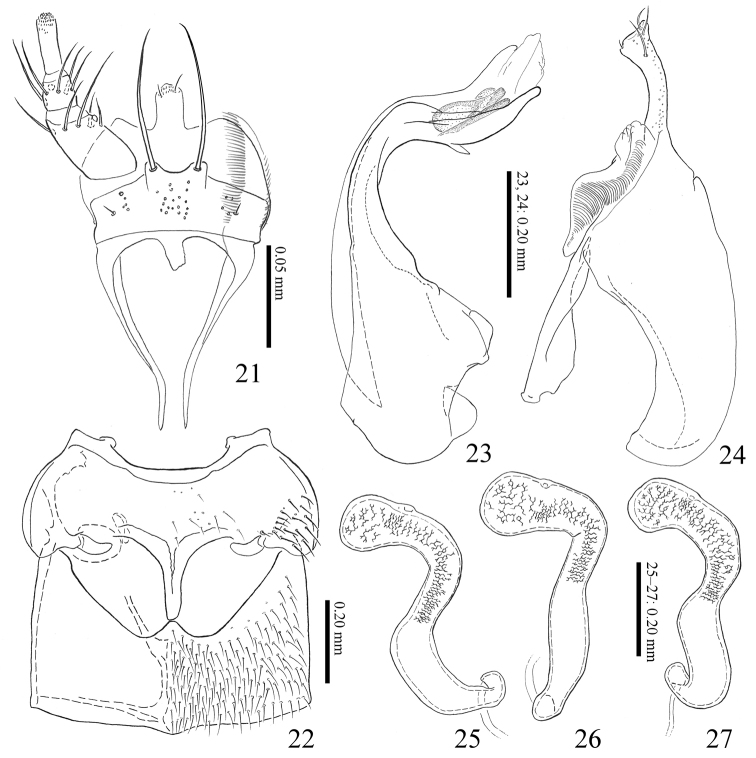
*Homoeusaovata* sp. nov. **21** labium **22** meso-, metaventrite **23** median lobe of aedeagus from lateral view **24** paramere of aedeagus from lateral view **25–27** spermatheca.

***Head*** (Fig. 17) relatively large, reddish brown; frontal margin with few long setae; carina of antennal insertion simply bent; eye large. Antennae (Fig. 18) stout, as long as head and pronotum combined, reddish brown, but segments I–III and XI paler; segment I dilated, widest near apex; segment II longer than III, widened apically; segment III widened apically; segment IV–X widened apically, each apical margin fringed with small teeth and longer than wide; segments IV as long as wide; segments V–X slightly wider than long; segment XI oblong oval. Labrum (Fig. 19), apical margin weekly concave surface with 20 setae; epipharynx with 2 pairs of sensillae on anterior margin, and 3 pairs of micro setae on lateral margin. Mentum (Fig. 20) with 3 pairs of setae and 1 pair of microsetae; anterior margin shallowly concave. Prementum (Fig. 21) with many pseudopores, and with 4–5 real pores and 1 setal pore on each side near middle lateral margin. Ligula elongate, apical margin with 1 pair of spinula and several sensilla. Labial palpus (Fig. 21), segment II and III each with 5 setae.

***Pronotum*** (Fig. 17) convex, subrectangular, strongly transverse, widest near middle, posterolateral angle obtuse, posterior margin hardly sinuate; brownish red to brownish black; surface finely covered with setae and punctures, gently polished. Elytra (Fig. 17) slightly widened posteriorly, posterior margins shallowly notched near lateral corners, brownish red; surface finely covered with setae and punctures, gently polished. Mesoventral (Fig. 22) processes narrow, with medial carina, forming Y-shaped, apex rounded; mesocoxal cavities almost separated; metaventral process gently produced.

***Abdomen*** elongate, gently narrowed posteriad; surface sparsely covered with short setae and each posterior margin with long stout setae; gently polished and weakly reticulated.

**Male**: 8^th^ sternite longer than wide, rounded apically. Median lobe of aedeagus (Fig. 23), apical lobe of male aedeagus s-shaped with small lanceolate flattened projection; apical lobe of paramere (Fig. 24) curved ventrally and thickened apicad with 4 setae gathering to apex; velum emarginated at middle; ventral margin of paramerite strongly concaved.

**Female**: 8^th^ sternite longer than wide, rounded apically. Spermatheca (Figs 25–27), apical part weakly swollen; apical half with inner wall densely reticulated.

##### Measurements.

Body shape (*N* = 20): BL ≈ 1.91–2.97; AL, 0.61–0.76; HW, 0.45–0.51; PL, 0.45–0.53; PW, 0.72–0.84; EL, 0.38–0.48; EW, 0.73–0.87; HTL, 0.47–0.53; PW/PL, 1.55–1.68; AL/PL, 1.26–1.68; HTL/PL, 0.93–1.12. Aspect ratio (length/width) of each antennal segment from I to XI (*N* = 6): 1.29–1.67, 1.29–1.75, 0.87–1.2, 0.72–0.82, 0.50–0.55, 0.43–0.50, 0.41–0.55, 0.33–0.61, 0.38–0.48, 0.41–0.54, 1.25–1.48.

##### Variation.

In most individuals, the pronotum tends to be darker, but the color varies from brownish red to brownish black.

##### Distribution.

Russia (Primorskyi krai), Korea, Japan (Hokkaido, Honshû, Shikoku, Kyûshû).

##### Bionomics.

This species behaves almost the same as *H.rufescens*. It is also observed to feed on prey among ants and sometimes climbs on, and eats food carried by ants (Fig. 31).

**Figures 28–31.**
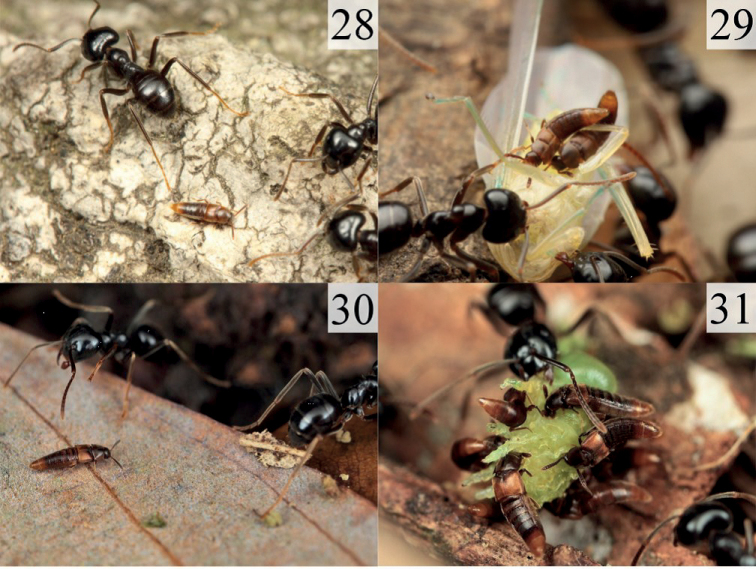
Photos of alive individuals of *Homoeusa* spp. in trails of *Lasiusfuliginosus* species group in field environments **28, 29***H.rufescens*: **28** waking on a trail of *Lasiusmorisitai***29** feeding on food being carried by ant workers (photographed by Taku Shimada) **30, 31***H.ovata* sp. nov.: **30** waking on a trail of Lasiuscf.fuliginosus**31** feeding on food being carried by ant workers (photographed by Kyoichi Kinomura).

##### Symbiotic hosts.

*Lasiusfuji*, L.cf.fuliginosus, *Lasiusmorisitai*, *L.nipponensis*, *L.orientalis*, *L.spathepus*.

## ﻿Discussion

As Quinet and Pasteels (1996) reported, *Homoeusaacuminata* has been observed to climb on prey being carried by ants and feed on it, simultaneously hitchhiking and stealing food. This beetle has also been reported to rarely feed directly on food not yet carried by ants (Quinet and Pasteels 1995). Similar behaviors have been observed in *H.rufescens* and *H.ovata* sp. nov., such as stealing food while boarding and being ignored by the ants during the process. However, *H.rufescens* and *H.ovata* sp. nov. have also been observed feeding on food that ants are trying to carry and on prey dropped by ants. These two species may have a wider range of foraging strategies than *H.acuminata* and may also scavenge and not merely be kleptoparasitic. The optimal foraging strategy for these beetles near an ant trail may depend on the level of ant activity.

### ﻿Checklist of the genus *Homoeusa* Kraatz, 1856

#### Palearctic

**1. *Homoeusaacuminata* (Märkel, 1842): 143.**

*Euryusaacuminata* Märkel, 1842

*Homoeusatomentosa* Reitter, 1909: 38

**Distribution.** Azerbaijan, Austria, Belgium, Belarus, Croatia, Russia (Central European Territory), Czech Republic, France, Great Britain, Germany, Georgia, Greece, Hungary, Italy, The Netherlands, Poland, Romania, Slovakia, Spain, Russia (South European Territory), Switzerland, Russia (Far East).

**Host.***Lasiusfuliginosus*.

**2. *Homoeusachinensis* Pace, 1999: 150.**

**Distribution.** China (Beijing).

**Host.** Unknown.

**3. *Homoeusajaponica* Sharp, 1874: 5.**

**Distribution.** Japan (Honshu, Shikoku, Kyushu).

**Host.**L.cf.fuliginosus, *L.morisitai*, *L.spathepus*.

**4. *Homoeusalaevigata* Sharp, 1888: 283.**

**Distribution.** Japan (Honshu).

**Host.***L.nipponensis*.

**5. *Homoeusalongicornis* Sharp, 1888: 283.**

**Distribution.** Japan (Hokkaido, Honshu).

**Host.**L.cf.fuliginosus, *L.morisitai*, *L.spathepus*.

**6. *Homoeusaovata* sp. nov.**

**Distribution.** Russia (Primorskyi krai), Korea, Japan (Hokkaido, Honshu, Shikoku, Kyushu).

**Host.***L.fuji*, L.cf.fuliginosus, *L.morisitai*, *L.nipponensis*, *L.orientalis*, *L.spathepus*.

**7. *Homoeusaprolongata* Sawada, 1970: 57.**

**Distribution.** Japan (Hokkaido, Honshu, Shikoku, Kyushu).

**Host.***L.japonicus*.

**8. *Homoeusarufescens* (Sharp, 1874): 5.**

*Thiasophilarufescens* Sharp, 1874

**Distribution.** Japan (Honshu, Shikoku, Kyushu).

**Host.**L.cf.fuliginosus, *L.morisitai*, *L.nipponensis*, *L.spathepus*.

**9. *Homoeusasibirica* Rambousek, 1921: 86.**

**Distribution.** Russia (Far East), Korea (South).

**Host.** Unknown.

**10. *Homoeusataiwanensis* Pace, 2010: 29.**

**Distribution.** Taiwan (Kaohsiung).

**Host.** Unknown.

#### Nearctic

**11. *Homoeusacrinitula* (Casey, 1900): 53**.

*Soliusacrinitula* Casey, 1900

*Homoeusafrosti* (Casey, 1911): 53

*Soliusafrosti* Casey, 1911

**Distribution.** America (New York, Massachusetts).

**Host.** Unknown.

**12. *Homoeusacrassicornis* (Casey, 1893): 595.**

*Myrmobiotacrassicornis* Casey, 1893

**Distribution.** America (Iowa).

**Host.** Unknown.

## Supplementary Material

XML Treatment for Homoeusa

XML Treatment for Homoeusa rufescens

XML Treatment for Homoeusa ovata
