# ﻿Corrigendum: Nozaki T, Maruyama M (2022) Taxonomy of *Homoeusa* Kraatz, 1856 (Coleoptera, Staphylinidae) from the East Palearctic: I. *Homoeusarufescens* (Sharp, 1874) and a new allied species. ZooKeys 1121: 39–58. https://doi.org/10.3897/zookeys.1121.85489

**DOI:** 10.3897/zookeys.1125.94925

**Published:** 2022-10-26

**Authors:** Tsubasa Nozaki, Munetoshi Maruyama

**Affiliations:** 1 Entomological Laboratory, Graduate School of Bioresource and Bioenvironmental Sciences, Kyushu University, Fukuoka 819-0395, Japan Kyushu University Fukuoka Japan; 2 The Kyushu University Museum, Fukuoka 812-8581, Japan The Kyushu University Museum Fukuoka Japan

We recently published the description of a new rove beetle species *Homoeusaovata* (Nozaki and Maruyama 2022). However, no holotype depository is indicated in the paper. This is mandatory after 1999 according to the current International Code of Zoological Nomenclature, and the new species name would be a nomen nudum and unavailable (ICZN 1999: Art. 16.4.2). In this corrigendum, we indicate the holotype depository and its etymology, which we did not include in the original paper. We thank Dr. Alfred F. Newton (Field Museum of Natural History, Chicago) for kindly pointing out this error.

***Homoeusaovata* Nozaki & Maruyama, sp. nov.**

**Type material. *Holotype*.** “Mt. Maruyama / Sapporo-shi / <Hokkaido, JAPAN> / 6. VI. 1988 / M. Maruyama leg. / trail of ants” (*LF*cf*F*). ***Paratypes*.** See, Nozaki and Maruyama (2022).

**Type depository.** The holotype is deposited at the Kyushu University Museum, Fukuoka, Japan.

**Etymology.** The specific epithet *ovata* refers to the oval body shape of this new species.
